# AGEISM EXPERIENCED AND EXPRESSED: MANIFESTATIONS ACROSS CONTEXTS AND TRANSGRESSORS

**DOI:** 10.1093/geroni/igad104.0200

**Published:** 2023-12-21

**Authors:** Alison Chasteen

## Abstract

Ageism was first defined more than fifty years ago, yet its expressions and effects on society continue today. In this symposium, we examine manifestations of ageism across contexts and transgressors in order to determine the effects of ageism in light of a pandemic as well as a post-pandemic world. Important social contexts in which ageism plays a part are examined, such as the workplace and COVID-19 vaccination. As well, efforts to reduce ageism through confrontation are investigated, particularly with respect to reactions to ageist behavior. Providing a window into ageist communication during the pandemic, Bascu and colleagues detail how ageism was expressed in social media regarding policies for COVID-19 vaccination. Delving into the factors that drive older workers’ decisions to remain or to leave an organization, both Lagacé et al and Swift investigate the role that perceived ageism plays. Lagacé and colleagues assess how older workers’ perceptions of being the target of ageism impacts their well-being, their psychological disengagement, and their intentions to leave their organization. Swift considers how experiences of ageism in the workplace predict older workers’ retirement intentions through reduced job satisfaction and lowered intrinsic motivation to work. Chasteen et al. test the impact of confronting ageism on perceptions of transgressors as well as how perceivers use characteristics of an older adult target as a normative cue for determining their reactions to ageist behavior. Taken together, these presentations illustrate the influence of ageism on vital life domains such as healthcare and the labor force.

